# PReS-FINAL-2100: Methotrexate treatment affects effector, but not regulatory T cells in juvenile idiopathic arthritis

**DOI:** 10.1186/1546-0096-11-S2-P112

**Published:** 2013-12-05

**Authors:** S Vastert, M Bulatovic-Calasan, R Scholman, M Klein, N Wulffraat, B Prakken, FV Wijk

**Affiliations:** 1 Pediatric Immunology, Wilhelmina Childrens'Hospital, University Medical Center Utrecht, Netherlands; 2 Pediatric Immunology, University Medical Center, Utrecht, Netherlands; 3 Pediatric Immunology, Wilhelmina Childrens Hospital, University Medical Center Utrecht, Utrecht, Netherlands

## Introduction

The balance between regulatory (Treg) and effector T cells (Teff) is crucial for immune regulation in juvenile idiopathic arthritis (JIA). How methotrexate (MTX), the cornerstone treatment in JIA, influences this balance *in vivo *is poorly elucidated.

## Objectives

To investigate quantitative and qualitative effects of MTX on Treg and Teff in JIA patients during MTX treatment.

## Methods

Peripheral blood samples were obtained from JIA patients at MTX start and 3 and 6 months thereafter. Treg numbers and phenotype were determined by flow cytometry and suppressive function in allogeneic suppression assays. Teff proliferation upon stimulation with anti-CD3, activation status and intracellular cytokine production were determined by flow cytometry. Effector cell responsiveness to suppression was investigated in autologous suppression assays. Effector cell cytokines in supernatants of proliferation and suppression assays and in plasma were measured by cytokine multiplex assay.

## Results

MTX treatment in JIA did not affect Treg phenotype and function. Instead, MTX treatment enhanced, rather than diminished, CD4+ and CD8+ T cell proliferation of JIA patients after 6 months of therapy, independent of clinical response. Effector cells during MTX treatment were equally responsive to Treg-mediated suppression. MTX treatment did not attenuate Teff activation status and their capacity to produce IL-13, IL-17, tnfα and ifnγ. Similarly to Teff proliferation, plasma ifnγ concentrations after 6 months were increased.

## Conclusion

This study provides a novel insight that MTX treatment in JIA does not attenuate Teff function but conversely, enhances T cell proliferation and ifnγ plasma concentrations in JIA patients.

## Disclosure of interest

S. Vastert Consultant for: Novartis, < 1000 euro's., M. Bulatovic-Calasan: None declared., R. Scholman: None declared., M. Klein: None declared., N. Wulffraat Grant/Research Support from: Abbvie, Roche, Consultant for: Novartis, Pfizer, B. Prakken: None declared., F. Wijk: None declared.

